# Long Life Family Study Shows Reduced Coronary Artery Disease Despite High Polygenic Hazard Scores

**DOI:** 10.1093/geroni/igaa057.685

**Published:** 2020-12-16

**Authors:** Mary Feitosa, Allison Kuipers, Mary Wojczynski, Lihua Wang, Thomas Perls, Kaare Christensen, Joseph Zmuda, Michael Province

**Affiliations:** 1 Washington University School of Medicine, St Louis, Missouri, United States; 2 University of Pittsburgh, Pittsburgh, Pennsylvania, United States; 3 Boston University School of Medicine, Boston, Massachusetts, United States; 4 University of Southern Denmark, Odense C, Syddanmark, Denmark; 5 Washington University School of Medicine, SAINT LOUIS, Missouri, United States

## Abstract

Polygenic hazard scores (PHS) for coronary artery disease (CAD) quantify individuals with age-specific genetic risk for CAD. We evaluated how well the PHS predict age at onset of CAD in the Long Life Family Study (LLFS; families selected for exceptional longevity), compared to the Family Heart Study (FamHS; random families and high CAD-risk families). LLFS contains 4572 European ancestry (EA) individuals from 581 families (age: 74.0 ± 14.3, range: 22-110 years). FamHS Random has 1806 EA individuals from 454 families (age: 56.2 ± 13.5, range: 22-91 years), while FamHS High CAD-risk has 2301 EA individuals from 553 families (age: 53.2 ± 12.8, range: 21-93 years). We generated the PHS from 176 published SNPs from GWAS for CAD (p< 5.0 x 10-8, r2< 0.2). In each of the extremes of the CAD PHS distributions (75%), Kaplan-Meier method showed that the LLFS presented significant delayed age at onset of CAD compared to FamHS (random and High CAD-risk: P<0.0001). A Cox proportional hazards regression model accounting for CAD age at onset, using family bootstrap (N= 1000) to correct for family relatedness, replicated these results. For example, in the top-25% CAD-PHS when comparing to FamHS high-risk, the LLFS CAD hazards ratio was 0.127 (95% CI: 0.099, 0.164). Our findings suggest that, while PHS captured some of the risk of CAD in LLFS, part of the predisposition remains to be determined. Other relevant factors, including additional genetic discoveries and lifestyle-environment influences are needed to fully determine CAD risk in extreme samples.

